# Methods for estimating human endogenous retrovirus activities from EST databases

**DOI:** 10.1186/1471-2105-8-S2-S11

**Published:** 2007-05-03

**Authors:** Merja Oja, Jaakko Peltonen, Jonas Blomberg, Samuel Kaski

**Affiliations:** 1 Department of Computer Science, University of Helsinki, P.O. Box 68, FI-00014 University of Helsinki, Finland; 2 Helsinki Institute for Information Technology, Laboratory of Computer and Information Science, Helsinki University of Technology, P.O. Box 5400, FI-02015 TKK, Finland; 3 Section of Virology, Department of Medical Sciences, Uppsala University, Academic Hospital, 751 85 Uppsala, Sweden

## Abstract

**Background:**

Human endogenous retroviruses (HERVs) are surviving traces of ancient retrovirus infections and now reside within the human DNA. Recently HERV expression has been detected in both normal tissues and diseased patients. However, the activities (expression levels) of individual HERV sequences are mostly unknown.

**Results:**

We introduce a generative mixture model, based on Hidden Markov Models, for estimating the activities of the individual HERV sequences from EST (expressed sequence tag) databases. We use the model to estimate the relative activities of 181 HERVs. We also empirically justify a faster heuristic method for HERV activity estimation and use it to estimate the activities of 2450 HERVs. The majority of the HERV activities were previously unknown.

**Conclusion:**

(i) Our methods estimate activity accurately based on experiments on simulated data. (ii) Our estimate on real data shows that 7% of the HERVs are active. The active ones are spread unevenly into HERV groups and relatively uniformly in terms of estimated age. HERVs with the retroviral *env *gene are more often active than HERVs without *env*. Few of the active HERVs have open reading frames for retroviral proteins.

## Background

Human endogenous retroviruses (HERVs) are surviving traces of ancient infections by retroviruses that have become fixed to human DNA. If ancient highly mutated elements are included, HERV sequences form 8% of the human genome [1].

HERVs are DNA sequences with a typical retroviral structure. A full-length HERV sequence is about 9,000 base pairs long and has a long terminal repeat (LTR) at each end. The rest, the internal part, of the HERV consists of 4 retroviral genes: *gag*, *pro*, *pol *and *env*. A functional, active HERV can transcribe its genes and produce retroviral proteins. These proteins enable the HERV to move and copy its DNA to other locations in the genome; such copying eventually yields several mutated versions of the original virus. A set of sequences mutated from the same virus is called a group; a group may contain hundreds of very similar sequences. A list of HERV groups can be found for example in [2].

Some of the HERVs have lost the typical retrovirus structure through mutations and various genomic rearrangements, and may contain mutated versions of only some or even one of the viral genes and/or zero to one LTRs. As a result, the present-day HERVs are mainly unable to move and copy themselves. Naturally, older elements have had more time to mutate and generally are less intact. The age of the retroviral element can be estimated from the sequence similarity of its two LTRs: they are identical upon integration and mutate afterwards.

HERVs are interesting for two reasons: they can express viral genes in human tissues, and their presence in the genome may affect the functioning of nearby human genes. Retroviral activity might cause disease; retroviral mRNAs have been detected in schizophrenia, autoimmune diseases and cancer [3], although a causal role of HERVs in these conditions is highly uncertain. On the other hand, a few retroviral genes have been co-opted for functions beneficial to the human host [4].

In this paper we analyze the activity of individual HERV sequences. Being able to do so is vital for analyzing their individual control mechanisms and their possible roles in diseased and normal cell functions. Most previous studies of HERV expression report activities only for HERV groups (e.g. [5]); the only exceptions we know of are [6] where a small test for individual HERVs of one group was done with a heuristic method and [7] where HERVs were searched from gene mRNAs but activities were not compared across HERVs.

To find evidence of HERV expression, we use a large public database of expressed sequence tags (ESTs) which are short and "noisy" mRNA samples. The amount of ESTs coming from a particular HERV is evidence of its activity (expression level). However, the noise level in ESTs can be larger than the sequence differences within a group, so it may be hard to determine exactly which HERV an EST stems from. This is the *EST matching problem*. Two HERVs whose ESTs are often confused are said to have *cross-talk*. In this work, we introduce a generative mixture model to model the uncertainty in the EST to HERV matching. The model learns the relative activities of the HERVs from EST sequence data. We validate the performance of our model with simulated EST data and then proceed to estimating the activities of HERVs. It turned out during our experiments that a fast heuristic method performed reasonably accurately on simulated data, which made it possible to analyze very large HERV collections.

## Methods

### Hidden Markov Mixture Model

We introduce a method for solving the EST matching problem. We start from the following assumptions about how ESTs are generated from HERVs: (i) EST transcription starts at some point of the HERV sequence; (ii) the EST sequence follows the HERV sequence, but can contain mismatches between the EST and HERV nucleotide, and can skip HERV nucleotides or insert new ones; (iii) lastly, the end of the EST sequence is of lower quality and does not resemble the HERV sequence.

We design a generative mixture model to mimic EST generation from HERVs, based on the above-mentioned assumptions. Each mixture component is a Hidden Markov Model (HMM) for ESTs from a particular HERV; such a HMM generates data that roughly matches a sub-sequence of the source HERV, but with mismatches, insertions, deletions, and a low-quality end part. The EST can match the HERV in either orientation. We need to make a small change to the model for those ESTs that match in reverse orientation. The modification is explained in the Additional file 1.

Hidden Markov mixture models have also been used in other bioinformatics applications, for example in [8] where HMM mixtures are used to cluster protein sequences, and in [9] where HMM mixtures were used to model gene expression time-courses.

**Figure 1:**
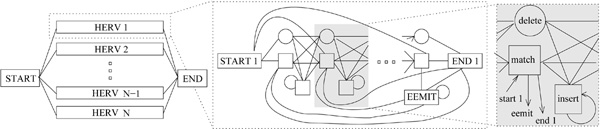
**The structure of the HMM mixture**. The structure of the HMM mixture is shown on the left. Each sub-HMM has the structure shown in the middle. The shaded box is the basic block of the sub-HMM and is repeated length-2 times. It is identical in all sub-HMMs; only the emission distribution of the match state varies between blocks. The emission is either the nucleotide in that position of the HERV sequence or a mismatch. The probabilities for match and mismatch are equal for all blocks. The EEMIT-state emits the low-quality end part.

The mixture can be interpreted as one large HMM where the first transition chooses one of the *N *HERV-specific sub-HMMs (see Fig. 1). We use the Baum-Welch algorithm to learn the whole mixture. The learned probabilities of the first transition (the mixture weights) are estimates of the HERV activities; learning the mixture model thus solves the EST matching problem. In addition to HERV activities, the best matches of individual ESTs to HERVs can also be computed from the learned HMM. However, we analyzed such matches only for large data where we use a simpler alternative; see the section *BLAST approach*.

We constrain the model complexity by sharing parameters. Each match state corresponds to a nucleotide in a HERV sequence; the probabilities of emitting the "correct" HERV nucleotide or a mismatch are the same for all match states. Other emission and transition parameters are shared between all the basic blocks (shown in Fig. 1) of all the sub-HMMs.

### Data

#### HERV data

We have 3164 individual sequences in our complete HERV data set. They were detected automatically from the human genome by the program RetroTector (Sperber, Blomberg et al, submitted); see the Appendix for details. RetroTector also annotates the HERVs; it estimates the structure of the element (presence and locations of LTRs and viral genes), the age of the element, the intactness of viral gene reading frames etc. It further classifies the HERVs into groups, based on sequence similarity to known representatives of the group. (Some sequences remain unclassified because they are not similar to the reference sequence of any group.)

#### EST data

We use two kinds of EST data: real and simulated.

Real ESTs matching the HERVs were searched from the dbEST database [10] with BLAST [11]. We first used a cutoff of E-value < 10^-25 ^in BLAST, and then removed all ESTs that match the HERVs in both orientations. This was done to remove suspicious ESTs; we assume that a retrovirus sequence does not contain long sequence portions that would match itself when reverse complemented. Then we used a strict match threshold of E-value < 10^-60 ^to get the final list of ESTs. We assume that the ESTs retrieved using the latter E-value threshold are of retroviral origin and do not match non-retroviral portions of the human genome, such as human genes. As a simplification, we kept only the best match for each EST-HERV pair; see the Additional file 1 for a discussion. Note that the ESTs in dbEST are measured from different tissues and conditions; in this paper we discuss average HERV activities, over the tissues and conditions in their proportions in the database. Tissue and condition-specific activities will be studied later.

For comparison studies we generated artificial ESTs from a set of HERVs using our HMM model. To make this simulated EST data as realistic as easily possible, the parameters of the generating HMM were set close to the parameters learned from real ESTs, and the lengths of the ESTs were controlled with a heuristic. After generating the ESTs we treated them following exactly the same procedure as with real data, starting with BLAST to match the HERVs against the ESTs.

#### Removing HERVs with suspected non-retroviral content

In some cases our detected HERV sequences may contain long stretches not annotated as any viral gene or LTR by RetroTector. It is a matter of choice whether to use the whole HERV sequence when searching for EST matches, or to only search with the sequences of each viral gene separately. Both approaches have their own merits; here we did the former.

A problem with our approach is that some of the un-annotated portions might in reality be non-retroviral. To control this phenomenon, we kept in our HERV set only those sequences where the real ESTs matched mainly in the annotated areas of the HERV sequence (85% of EST hit mass overlaps the viral genes or LTRs); we removed the other sequences from the HERV set. This removal leaves out HERVs where the EST matches are mainly in suspected non-retroviral areas.

Note that our approach enables us to find ESTs where the match overlaps multiple viral genes, or where the match overlaps both a viral gene and an un-annotated area. This would not have been possible if ESTs had been searched with separate sequences for each viral gene.

#### Three data sets

We used three different data sets in our experiments:

##### A small well-chosen set

First, we used a small set of 181 sequences from three HERV groups: HERV-W, HML-2, and HERV-E. The groups were selected based on previous studies where they were reported to be active (e.g. [5,12]). We used this subset of HERVs as an example of HERV data. We estimated the activities of these HERVs from real EST data using our model: the dbEST database yielded 1988 ESTs that match at least one HERV, with on average 118 EST matches per HERV.

##### A small simulated set

The same set of 181 HERVs was used to generate simulated EST data. The parameters of the generating HMM were set equal to those learned from real ESTs above, with small random deviations introduced. We generated 2500 ESTs, yielding 2012 ESTs that match at least one HERV, with on average 148 EST matches per HERV. The reason why some ESTs were discarded is that they have too long low-quality portions and the longest match in the BLAST step will then be short compared to the EST length, resulting in a large E-value. This simulated data was used to estimate the amount of cross-talk between HERVs, and the performance of our model.

##### Full set

Lastly, we estimated the activities for all HERVs in our data set, using real EST data. We originally had 3164 HERVs and 44653 real ESTs, with on average 42 EST matches per HERV. After sequences with suspected non-retroviral portions were removed, 2450 HERVs with 9393 real ESTs were left.

### HMM training time

The computational complexity of the HMM model is relative to the product of the following: number of iterations, HERVs and ESTs, and total lengths of the HERVs and the ESTs.

We make the HMM training time reasonable by applying two shortcuts. (i) Only HERV-EST pairs returned by BLAST are used (others get zero probability). (ii) We introduce the restriction that the EST can only match the HERV sequence in the immediate vicinity of the BLAST match. We tested the effect of the shortcuts on a tiny test data set; the shortcuts gave (with a very high precision) the same results as the complete model (results not shown). The computational complexity is reduced to the product of number of iterations, number of HERV-EST pairs, and squared average length of the match area.

### BLAST approach

A straightforward alternative to the HMM mixture is to neglect any cross-talk between the HERVs. Their activities can then be estimated simply by the number of BLAST hits. Each EST is counted in favor of its best matching HERV and the activities of HERVs are given as counts of ESTs in their favor ("EST hits"). We investigated with simulated data whether this computationally much more feasible method would be accurate enough; see the section *Simulated data *below. A similar BLAST approach was used in [6] for a tiny data set containing only intact HERV sequences.

### Reliability estimation by resampling

The BLAST approach produces an activity distribution over HERVs. The reliability of the distribution can be estimated with a bootstrap-like method as follows: The EST data is resampled with replacement several times (here 10,000 times) and the EST counts are recomputed for each replicate. See Supplementary Fig. 7 in Additional file 1 for details. A similar approach could be used to estimate the reliability of the activity distribution obtained with the HMM method; activities are reoptimized for each replicate while other parameters are kept fixed.

We compute a threshold value for activity as follows. For each HERV, we compute the 95% confidence interval for its activity value from the bootstrap samples for the BLAST approach. We then find the minimum EST count such that if the EST count of a HERV is at least this value, then zero (inactivity) is not included in its confidence interval. For the full HERV data this threshold value is 5 EST hits; in our results we call HERVs with at least 5 hits active.

## Results and discussion

### Simulated data

Sequences within a HERV group are very similar in sequence, and the differences are larger between groups. As a result, there is more cross-talk between sequences of the same group than between groups. We can directly estimate the amount of cross-talk in our simulated data by measuring how many ESTs that were originally generated from one HERV match another HERV. We observe more cross-talk in HML-2 than in the other two groups. HML-2 is more difficult from the point of view of the EST matching problem. See Supplementary Fig. 1 in Additional file 1 for details.

**Figure 2:**
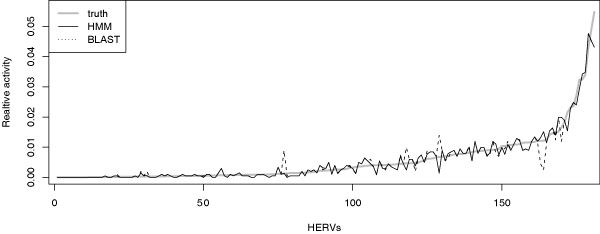
**Performance evaluation of HERV activity estimation on simulated data**. Activity distributions from simulated data. The activities learned by the HMM mixture and the simple BLAST approach are compared to the true generating distribution. The HERVs on the x-axis have been sorted according to relative activity in the true generating distribution.

The HMM method performed slightly better than the BLAST approach. The Kullback-Leibler divergence between the learned activity distribution and the generating distribution (ground truth) was 0.045 for the HMM method and 0.065 for the BLAST approach; see Fig. 2. As would be expected, the difference is a bit larger for the HML-2-elements that have more cross-talk between elements. The HMM model preserved 26 and BLAST 24 of the top 30 most active HERVs. The top 30 HERVs cover about 60% of the generating activity distribution. The simple BLAST approach is surprisingly good compared to HMM-based modeling; this suggests that it can be used for large tests where HMM training would be computationally too costly.

### Real data

#### Overview of HERV activity

We estimated the activities for the large set of 2450 HERVs. To save time the activities were estimated using the fast BLAST-based approach. About 7% (165) of the HERVs were active, that is had at least 5 EST hits in their viral gene or LTR areas. However, 10 of the most active HERVs explain as much as 60% of the activity distribution and have over a hundred EST hits. Most of the HERVs are inactive based on the EST collection used; 1903 HERVs had no EST matches. We estimated the reliability of the results with the bootstrap method and set the threshold for activity so that the confidence interval for active HERVs does not include zero (see section *Reliability estimation by resampling*). Thus we can reasonably trust the active ones to truly be active.

#### Relationship between HERV structure and activity

**Figure 3:**
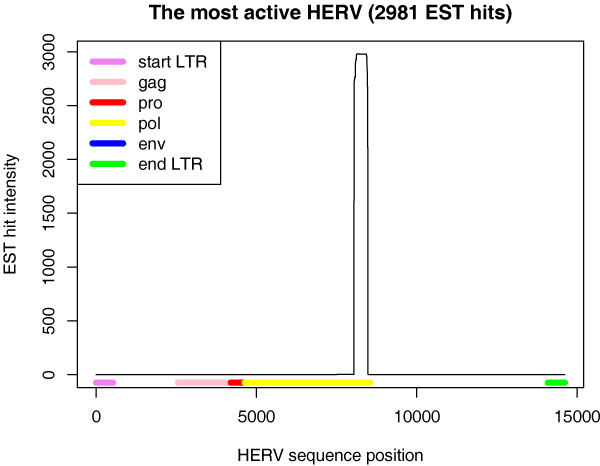
**EST hit location for the most active HERV**. The colored blocks below the curve represent the HERV structure (genes, LTRs) and the curve presents EST hit intensity along the HERV structure. See Table 1 for more information on this HERV. EST hit areas for other highly active HERVs are shown in Supplementary Fig. 4 in Additional file 1.

There are several kinds of HERVs among the active ones; old and young, full-length and those missing several viral genes, HERVs with open reading frames in their genes and HERVs that do not code for viral proteins, HERVs from almost all HERV groups. The EST sequences match various portions of the HERVs. In some cases the ESTs match (portions of) one or several of the viral genes, in some cases none. See Fig. 3 and Supplementary Fig. 4 in Additional file 1 for examples.

We explored the correlation of HERV activity to various annotations with an exploration set (or "training set"; half of the HERVs). The observed effects were verified with an independent test set (the rest of the HERVs).

**Figure 4:**
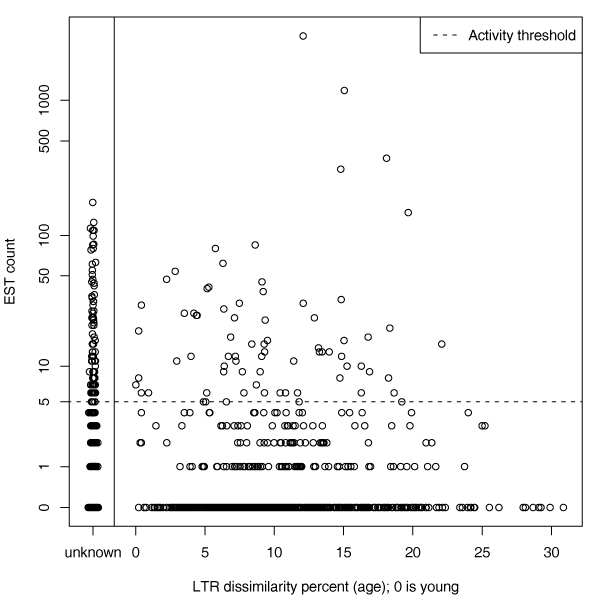
**Correlation between estimated age and activity**. Estimated age vs. activity (EST count) plot for all HERVs. HERVs with unknown age are plotted separately on the left (random jitter has been added in the age direction). We can see that there is no clear correlation between estimated age and activity. There is a more detailed figure in the Additional file 1.

We had expected that young elements would be more active and that the age of the element would correlate negatively with activity. For elements that have both LTRs, we were able to estimate the age of each element from LTR dissimilarity and check the hypothesis. However, it turns out active HERVs can also be found among the old elements. Fig. 4 shows that the age does not correlate with transcriptional activity. Another hypothesis is that the presence of an LTR in the beginning of the HERV sequence could itself explain activity, because the LTR contains transcription factor binding sites for human transcription factors. The LTR is basically designed to activate the viral genes. However, this hypothesis cannot explain all activity since an LTR in the beginning was not detected for almost half of the active elements.

The data shows, somewhat unexpectedly, that HERVs having an *env*-gene are more often active (13% are active) than those without an *env*-gene (only 4% are active). The difference is significant (Wilcoxon rank sum test *p *< 10^-11^). See Supplementary Fig. 3 in Additional file 1 for a closer view of the difference. The presence or absence of the other viral genes seems to have no clear effect. This suggest that expression from *env*-containing elements has had selective value. Note, however, that it is not necessarily the *env*-gene area that is active in the *env*-containing HERVs. In fact, in only about a third of the *env*-containing HERVs do the EST hits overlap the *env*-gene to some extent.

The data suggests that in many cases the retroviral sequence has been used as a building block for something else than retroviral proteins, for example human gene exons, promoters or polyadenylation signals. The evidence for this is: (i) only a few of the active elements have viral gene open reading frames, and (ii) the ESTs often match only a short portion of a viral gene (See Fig. 3 and Supplementary Fig. 4 in Additional file 1). However, for some HERVs there may be an alternative explanation: the retroviral transcripts may have RNA-mediated activities.

#### Active HERV groups

**Figure 5:**
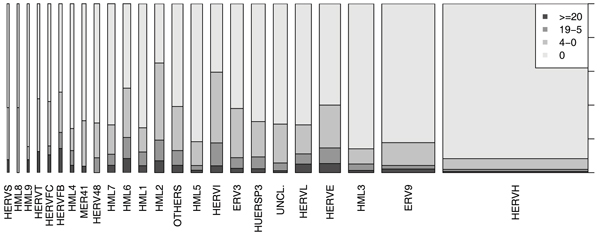
**Proportion of active HERVs in the groups**. The proportion of active HERVs in each group is presented with a stacked area plot. The two darkest gray areas together show the proportion of active HERVs in that group, the lightest gray area shows the proportion of inactive HERVs. The widths of the bars are proportional to the size of the HERV group. We can see that the proportion of active and inactive HERVs varies a lot from group to group.

Almost all groups have some active elements. However, the proportion of active HERVs varies greatly from group to group, see Fig. 5. For example, in HERV-FB, HML-6, HML-2 and HERV-I about 20% of the HERVs are active, whereas in the largest group HERV-H only 2% are active. Another interesting aspect is the proportion of totally inactive HERVs. The young HML-2 group has very few of these. An explanation for this may be the large amount of cross-talk in HML-2; some of the EST hits for the almost inactive HERVs (1 to 4 EST hits) may actually be coming from other active HERVs. On the other hand, in HERV-H about 90% of the elements are inactive. This is curious because the group is so large that it must have been able to actively copy its members at some stage (usually proliferation happens through activation of the moving element). There is evidence, however, that the proliferation of HERV-H has happened with the help of so called "midwife" elements that have copied the inactive HERV-H elements [13].

The HERV group activity can be summarized by accumulating the EST counts of its members. We compared the summed activities to earlier expression studies where group level activities were reported [5,6,12,14,15]. The groups reported as active were found to be among the most active also in our studies and, vice versa, the inactive groups were among the less active groups in our results. This comparison partly validates our approach. The summed activities are shown in Supplementary Fig. 6 in Additional file 1.

#### Cross-talk with undiscovered HERVs

It is not always possible to take all HERVs into the HERV set, for example because of limited computational time or because some HERVs are yet undiscovered. This naturally affects the group activity levels, but also the activities of the individual HERVs in the HERV set: some ESTs matching the HERVs might match an un-included HERV better.

**Figure 6:**
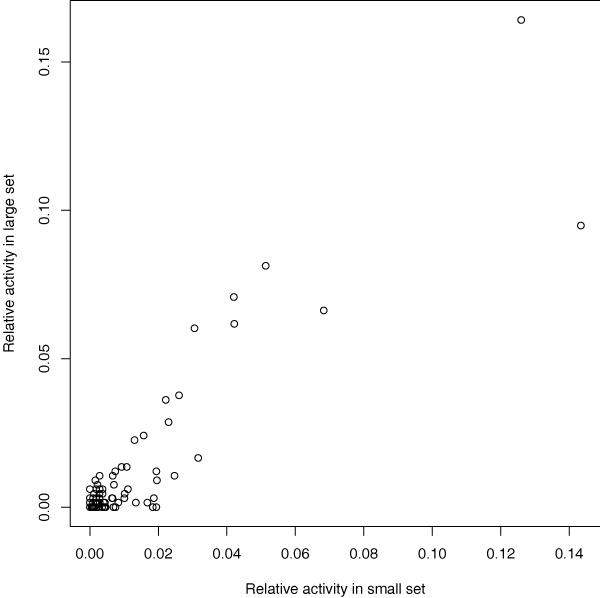
**Effect of cross-talk with undiscovered HERVs**. Comparison between results from a small subset of 181 HERVs (learned with the HMM mixture model) and results from the complete set of 2450 HERVs (learned using the BLAST approach). The scale of the figure is such that the relative activities for the HERVs sum up to 1 in both x and y dimensions.

The possible error can be estimated by comparing the results estimated with the complete set of HERVs to results estimated with the smaller subset of 181 HERVs (with real ESTs for both sets). Optimally, the activity order of the 181 HERVs would remain the same when more HERVs are introduced to the model. Fig. 6 shows that HERVs that were detected as most active in the smaller set are still among the most active in the larger set. However, their rank order changes slightly between the sets.

#### Individual active HERVs

In this section we discuss some individual interesting HERVs that were detected as active by our method. We analyze in more detail both HERVs that were known to be active based on previous studies and also interesting highly active HERVs that were not previously known to be active. Table 1 summarizes the top 10 most active HERVs and two other sequences analyzed here.

The most active element is active in an area that is conserved in chimpanzee, mouse, and rat according to the UCSC Genome Browser. The ESTs match it in the end of the *pol*-gene (Fig. 3). The element is presumably old; it has 12% dissimilar LTRs.

In the second most active element the active area, located in the the *pol-env *gene border (Supplementary Fig. 4 in Additional file 1), is conserved in chimpanzee, mouse, rat, and chicken. The EST match area is also a predicted gene area at UCSC. The element is presumably old; it has 15% dissimilar LTRs. For the above two HERVs, the LTR dissimilarity translates to a time since integration of 30–40 million years ago (assuming 0.2% substitution per million years of a selection neutral sequence for each LTR). For the second most active HERV this is odd because the split between human and chicken was considerably earlier. The reasons for the conservation of the active areas are unknown.

The sixth active sequence acts as the ending point for a human gene. The sequence is presumably an old element (LTRs 19% dissimilar). The ESTs match the LTR in the beginning of the sequence (Supplementary Fig. 4 in Additional file 1). UCSC Genome Browser reveals that the LTR acts as the untranslated region at the end of a human gene, YY1AP1. This is natural, because the LTRs contain the polyadenylation signal sequence also used by the viral genes themselves.

The seventh active sequence has EST hits along its whole sequence (Supplementary Fig. 4 in Additional file 1). UCSC Genome Browser shows a gene prediction and mRNAs mapped to this location. This sequence is a potential candidate for a retrovirally active HERV. However, its viral gene reading frames are not open.

Both *syncytin *genes are detected as active. *Syncytins *are human genes derived from retroviral *env*-genes that have fusogenic functions in the placenta. 40 ESTs match *syncytin *HERV on all viral genes, not only on the *env*-gene. *Syncytin-2 *HERV has 20 EST matches that all overlap with the *env*-gene as would be expected. See Supplementary Fig. 5 in Additional file 1 for EST hit histograms.

The examples above show that our method is able to give detailed information about individual HERVs. In addition to these examples, the data includes several potentially interesting HERVs that merit further study.

#### Activity of group reference sequences

HERV activity is commonly studied for groups instead of individual HERVs; this is sometimes done by measuring the activity of hand-picked reference sequences for the groups. It is striking that when we looked at the activities of three reference sequences two of them were not the most active elements within the group (HML-2 and HERV-E). The reference sequence of HERV-W (subgroup of ERV-9 [16]) was the most active element in the group. All these reference sequences were active. These results suggests that the more active HERVs could be better probe sequences in expression studies.

#### Effect of leaving out HERVs with suspected non-retroviral sequence portions

As mentioned in the *Data *section, we removed from our HERV data those sequences where most of the EST matches were to an un-annotated portion of the sequence, because such matches might be signs of non-retroviral activity. What if we had not removed any sequences from the HERV set? Briefly, the main conclusions would not change, but the activity distribution of the HERV groups would show large changes. We believe that removing the sequences with suspected non-retroviral activity made our HERV set more relevant for analysis of HERV activity, but note that in some of the removed sequences, the un-annotated portions could on close inspection turn out to be retroviral after all. See the Additional file 1 for more details.

## Conclusion

We have introduced a generative model-based method that estimates the activities of individual HERVs rather than only HERV groups. Such detailed analysis is vital for understanding the underlying control mechanisms of HERV activation. HERVs reported as active with our method can later be verified with laboratory methods; by contrast, exhaustive search of active HERVs with laboratory methods would be too expensive.

In simulated data both the HMM mixture and a heuristic BLAST-based alternative were able to estimate underlying activities fairly well: the most active HERVs in the ground truth were among the most active in the results of both methods. This justifies the use of the computationally simpler alternative instead of the rigorous probabilistic method.

We were able to get a detailed picture of HERV activity in real data. Below we briefly summarize our main results so far.

In almost all HERV groups we detected one or several new active HERVs that need further biological analysis; altogether 165 HERVs. Overall, only 7% of the elements were active and more than two thirds of the HERVs were completely inactive. Various kinds of HERVs are included in the set of active HERVs (young, old, full-length, non-full-length). HERVs with the *env*-gene were observed to be active more often than sequences without *env*. On the other hand, no clear relation between age of the element and activity was visible.

The data suggests that in many cases the retroviral sequence has been used as a building block for something else than retroviral proteins, for example human gene exons. However, for some HERVs there may be an alternative explanation: the retroviral transcripts may have RNA-mediated activities. We need to study the active elements more closely to discover the possible functions of retroviral transcripts.

Our results are in accordance with earlier results on HERV activation. We observe activity/inactivity of the same groups as in earlier publications. Furthermore, we detect the two well known examples of active HERVs, the *syncytin *genes, as active. These observations support the claim that we can truly find real activities with our approach.

The generally used reference sequences (the first published sequence which is used to define a group) of the groups were not always the most active, which suggests that the more active HERVs could be better probe sequences in expression studies.

A possible future application is to study the interplay between the definition of the HERV groups and their activity. This can be done interactively, because the activities are computed in silico.

The proposed method is general; it can be used to compare HERV activities in different conditions, or to study endogenous retroviruses in other organisms, or to include other kinds of transposable elements.

## Authors' contributions

MO, JP and SK jointly designed the study and developed the HMM model. JB gave the initial idea of studying HERV expression and provided the HERV data. MO implemented the HMM model, collected the EST data, ran all the experiments and conducted most of the analysis of the results. JP, JB and SK took part in analyzing the results. All authors participated in writing the manuscript. All authors read and approved the final manuscript.

## Appendix

### RetroTector

The program package RetroTector (Sperber, Blomberg et al, submitted) is an expert system for identification of potential LTRs and ERVs. It rests on known data about conserved motifs and distances between them, combining hits by the motifs into chains fulfilling the distance constraints. Chains are assigned a score depending on number and goodness of hits, and a genus depending on the genus affiliation of the motifs. Chains with a good enough score are further analyzed, attempting to reconstruct the coded proteins, improving the LTRs etc.

HERV-L and HERV-S are underrepresented in the RetroTector data set. This is known by comparison of RetroTector data with RepeatMasker output and HERVd for the human genome hg15 assembly (Blomberg and Sperber, unpublished).

## Supplementary Material

Additional File 1 **Supplementary material**. contains supplementary details on our methods and supplementary figures 1-7. Click here for file

## Figures and Tables

**Table 1:** Selected highly active HERVs.

rank	chr	start	end	subgenes	group	ESTs	hit area
1	5	52510446	52525065	LTRgagpropolLTR	HERV-48	2981	(short) pol
2	2	98059068	98067632	LTRgagpropolenvLTR	ERV-9	1182	pol, env
3	5	180072600	180059089	LTRgagLTR	UNCL.	373	gag
4	2	62302244	62290382	LTRpropolLTR	UNCL.	310	pol
5	21	37885010	37876409	LTRgagpol	HERV-H	176	(short) pol
6	1	152846189	152836852	LTRgagpolenvLTR	MER-41	148	5' LTR
7	16	25125181	25118816	gagpropolenv	HERV-E	125	overall
8	3	8690554	8695505	gagpropol	HERV-E	113	pol
9	6	123050520	123043680	gagpropolenv	HERV-E	109	(short) pol
10	14	23890064	23879831	LTRgagpol	HERV-H	109	pol

31	7	91719541	91709354	LTRgagpropolenvLTR	ERV-9	40	all genes
62	6	11220436	11211151	LTRgagpropolenvLTR	HERV-FRD	20	env

Details about the 10 most active HERVs and the two HERVs that code for the syncytin proteins. The HERVs are sorted in a decreasing activity order, rank is the position in this ordering. "Chr", "start" and "end" tell the chromosome and sequence start and end positions of the HERV in this chromosome (in the July 2003 version (hg16) of the human genome). "Subgenes" describes the structure and "group" the HERV group of the HERV. "ESTs" is the count of EST matches to the HERV. "Hit area" briefly describes where the ESTs match the HERV. The HERV with rank 31 is *syncytin *and the HERV with rank 62 is *syncytin-2*.
