# Supplementary material for: Methods for estimating human endogenous retrovirus activities from EST databases
Source: BMC Bioinformatics. 2007 May 3;8(Suppl 2):S11. doi: 10.1186/1471-2105-8-S2-S11 (PMC1892069; doi:10.1186/1471-2105-8-S2-S11)

Supplementary material for: "Methods for estimating human endogenous retrovirus activities from EST databases"

Merja Oja*[1,2], Jaakko Peltonen[2] , Jonas Blomberg[3] and Samuel Kaski[2]

[1]Department of Computer Science, University of Helsinki, P.O. Box 68, FI-00014 University of Helsinki, Finland
[2]Helsinki Institute for Information Technology, Laboratory of Computer and Information Science, Helsinki University of Technology, P.O. Box 5400, FI-02015 HUT, Finland
[3]Section of Virology, Department of Medical Sciences, Uppsala University, Academic Hospital, 751 85 Uppsala, Sweden

Email: Merja Oja*- merja.oja@tkk.fi; Jaakko Peltonen - jaakko.peltonen@tkk.fi; Jonas Blomberg - jonas.blomberg@medsci.uu.se; Samuel Kaski - samuel.kaski@tkk.fi;

*Corresponding author

Supplementary details about our methods

HMM model and EST-data

Some EST sequences match the HERV sequence in reverse orientation because the reverse complement of the mRNA is sequenced instead of the forward copy. To account for this phenomenon, when computing the likelihood of such an EST sequence with the HMM model, the HERV sequence corresponding to the match states is also reversed in each sub-HMM. That is, the first match state in a sub-HMM has a high probability of generating an EST nucleotide matching the last nucleotide of the HERV sequence associated with the sub-HMM, the second match state has a high probability of generating a match to the second-to-last HERV nucleotide, and so on. This way, the HMM structure mimics the EST generation even for the reverse orientation ESTs; in particular, the low quality end part is used for the correct end of the EST sequences. In addition, the sequence is reverse complemented in the EST before it is given to the HMM (A→T, T→A, C→G, G→C).

Discussion on keeping only the best match for each EST-HERV pair

When querying for EST data from the dbEST database, we kept only the best BLAST match for each EST-HERV pair. Keeping only the best match affects our methods in three ways:

1. In the HMM training, the sub-HMM corresponding to the HERV generates the whole EST sequence: the match is only used to restrict the generation (the possible paths through the sub-HMM).

Including several alternative match areas could allow more freedom for the sub-HMM in the generation of the EST, which could yield a larger likelihood for the observed EST being generated from the sub-HMM, and hence a larger activity estimate for the corresponding HERV.

2. In the simple BLAST approach, each EST is counted in favor of its best-matching HERV; it does not matter how many matches to that HERV there are. Thus, the BLAST approach for activity estimation is not affected by how many matches per EST-HERV pair are kept.

3. Having several EST-HERV matches would have affected the estimate of the active areas of the HERV sequence (the activity would likely be spread more evenly).

Note that having more than one match for an EST-HERV pair would not increase the 'weight' of the EST in the activity estimation of the HERVs, since each EST is biologically generated only once. Rather, several matches indicate more uncertainty about where in the HERV the EST was generated from.
We briefly discuss three prototypical cases of multiple matches for the same EST-HERV pair:

1. If almost consecutive areas of the EST match almost consecutive areas of the HERV, the set of matches is almost the same as a single long match. In this case it is nearly equivalent to take the best one of the matches and use it in HMM training, because the training restrictions derived from the different matches are nearly equivalent.

2. If the same area of the EST matches several far-off areas of the HERV, only one match is true. If one of the matches is clearly best, the others are likely false; then keeping the best match only may reduce noise. If several matches are nearly equally good, keeping only the best may cause a small amount of error for activity estimation.

3. If consecutive areas of the EST match far-off areas of the HERV, this could be because of for instance alternative splicing. The HMM method and the BLAST approach do not currently take alternative splicing into account.

Details about leaving out HERVs with suspected non-retroviral sequence portions

Below we first discuss one reason for non-retroviral sequence portions; then we describe how our HERV removal procedure (that we used to try to remove effects of non-retroviral content) affects the HERV activity results.

Non-retroviral integrations in HERV sequences

Some HERV sequences in our collection could contain non-retroviral transposon integrations. To avoid such integrations, a rather stringent removal of specific transposons (ALUs and LINEs) was done before running RetroTector, but it is possible other non-retroviral integrations remain.

An even more stringent removal could be done by removing all transposons not indicated as retroviral in RepBase [1] and RepeatMasker [2]. However, this assumes these sources contain complete knowledge of which transposons are retroviral; in reality, HERVs not named as such in these sources could inadvertently be removed.

For the above reasons, a small occurrence of non-retroviral integrations in HERV sequences are in practice unavoidable.

Activity comparison with and without HERV removal

In this paper, as described in the section *Removing HERVs with suspected non-retroviral content*, we have tried to remove the effect of suspected non-retroviral content on HERV activity by leaving out from our HERV set HERVs with EST hits mostly in un-annotated portions of the sequence.

We compared the group summarized activity reported for the set of 2450 HERVs used in this work (See Supplementary Fig. 6) to that computed from all 3164 HERVs, i.e. including also HERVs where the activity is not within a viral gene or LTR. The activity profile changes quite a lot because then also HML-5, ERV-3, and MER-41 have highly active elements. HERV-H has several highly active elements and is the most active group in the complete set of 3164 HERVs, the second most active group is the unclassified sequences.

We think the activity distribution for the data set where HERVs with EST hits mostly in un-annotated portions of the sequence are removed is more relevant to analyzing real retroviral activity than the distribution for the data set of all HERVs. However, there can be some retroviral expression included in the set of HERVs that was left out: their expression might be in an area that is originally retroviral but has been left un-annotated because of mutations and frame-shifts.

References

1. Jurka J: **Repbase Update: a database and an electronic journal of repetitive elements**. *Trends in genetics* 2000, **16**(9):418–420.
2. Smit AFA, Hubley R, Green P: **RepeatMasker Open-3.0.** 1996-2004. [Http://www.repeatmasker.org].

3. Seifarth W, Frank O, Zeifelder U, Spiess B, Greenwood AD, Hehlmann R, Leib-Mösch C: **Comprehensive Analysis of Human Endogenous Retrovirus Transcriptional Activity in Human Tissues with a Retrovirus-Specific Microarray**. *Journal of Virology* 2005, **79**:341–52.

4. Stauffer Y, Theiler G, Sperisen P, Lebedev Y, Jongeneel CV: **Digital expression profiles of human endogenous retroviral families in normal and cancerous tissues**. *Cancer Immunity* 2004, **4**(2).

Supplementary Figures

Supplementary figure 1 - Amount of cross-talk in HERV data

The number of simulated ESTs generated from each HERV (row) that match the other HERVs (column) shown in logarithmic scale. The blocks in the diagonal correspond to the three groups in the data set. The generating HERVs are sorted block-wise by underlying true activity.

We can see that the HML2 has the most cross-talk. This group is more difficult from the point of view of the EST matching problem.

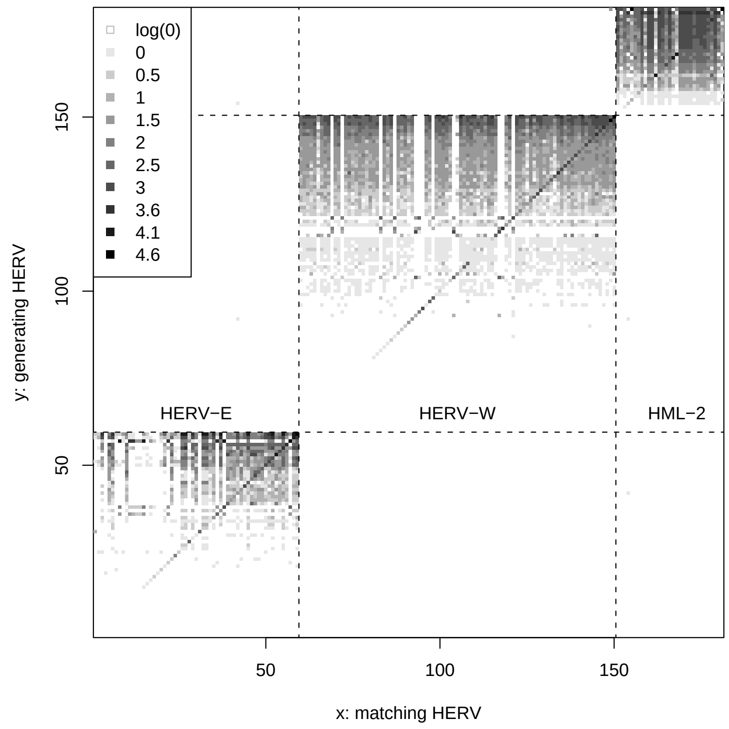

Supplementary figure 2 - Correlation between estimated age and activity

Estimated age vs. activity (EST count) plot for all HERVs. HERVs with unknown age are plotted separately on the left (random jitter has been added in the age direction). We can see that there is no clear correlation between estimated age and activity.

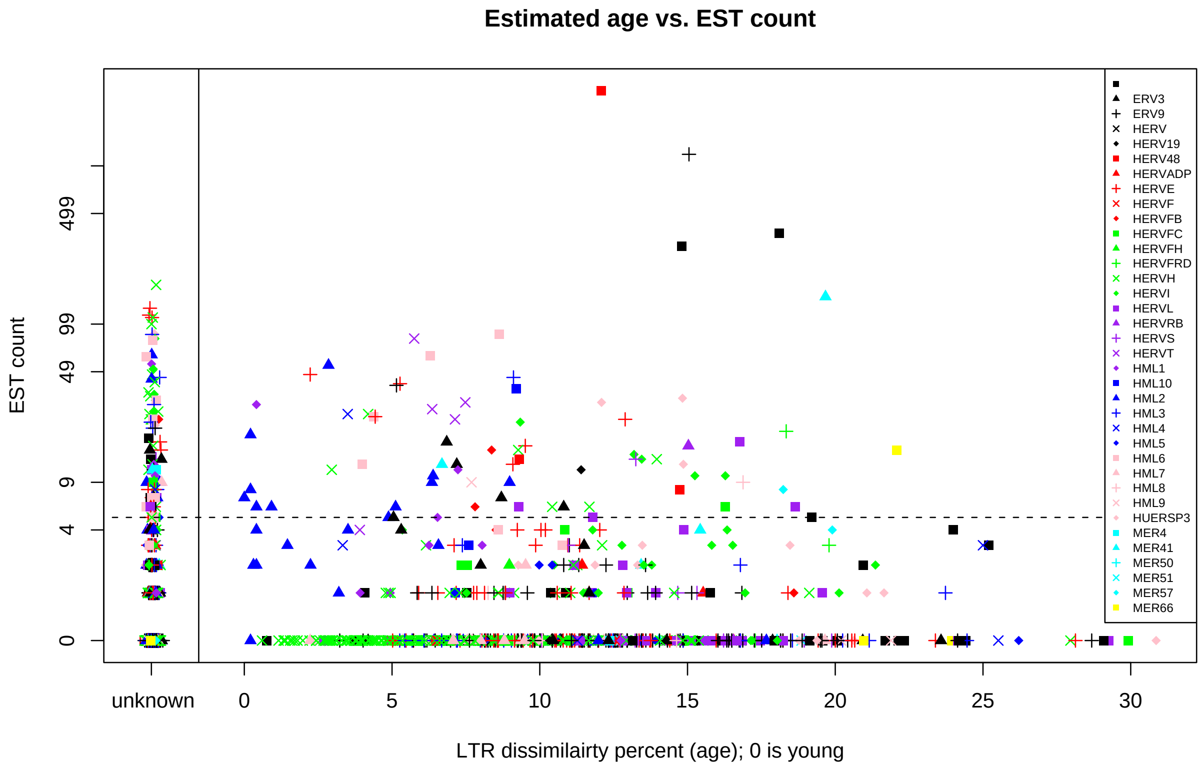

Supplementary figure 3 - Activity of HERVs with or without the env-gene

Proportion of active HERVs with different activity thresholds, for HERVs with and without the *env*-gene.

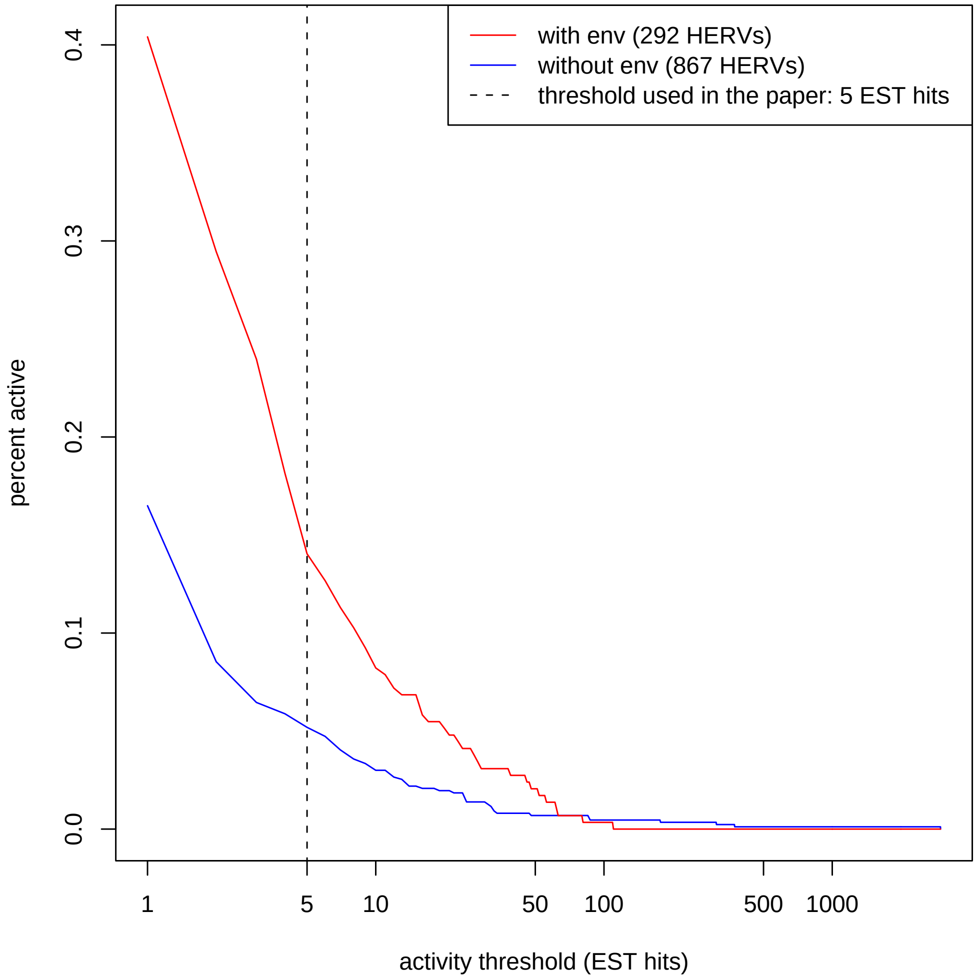

Supplementary Figure 4 - EST hit locations for the 10 most active HERVs

The colored blocks below the curve represent the HERV structure (genes, LTRs) and the curve presents EST hit intensity along the HERV structure. See Table 1 for more information on these HERVs. (Continued on the next page.)

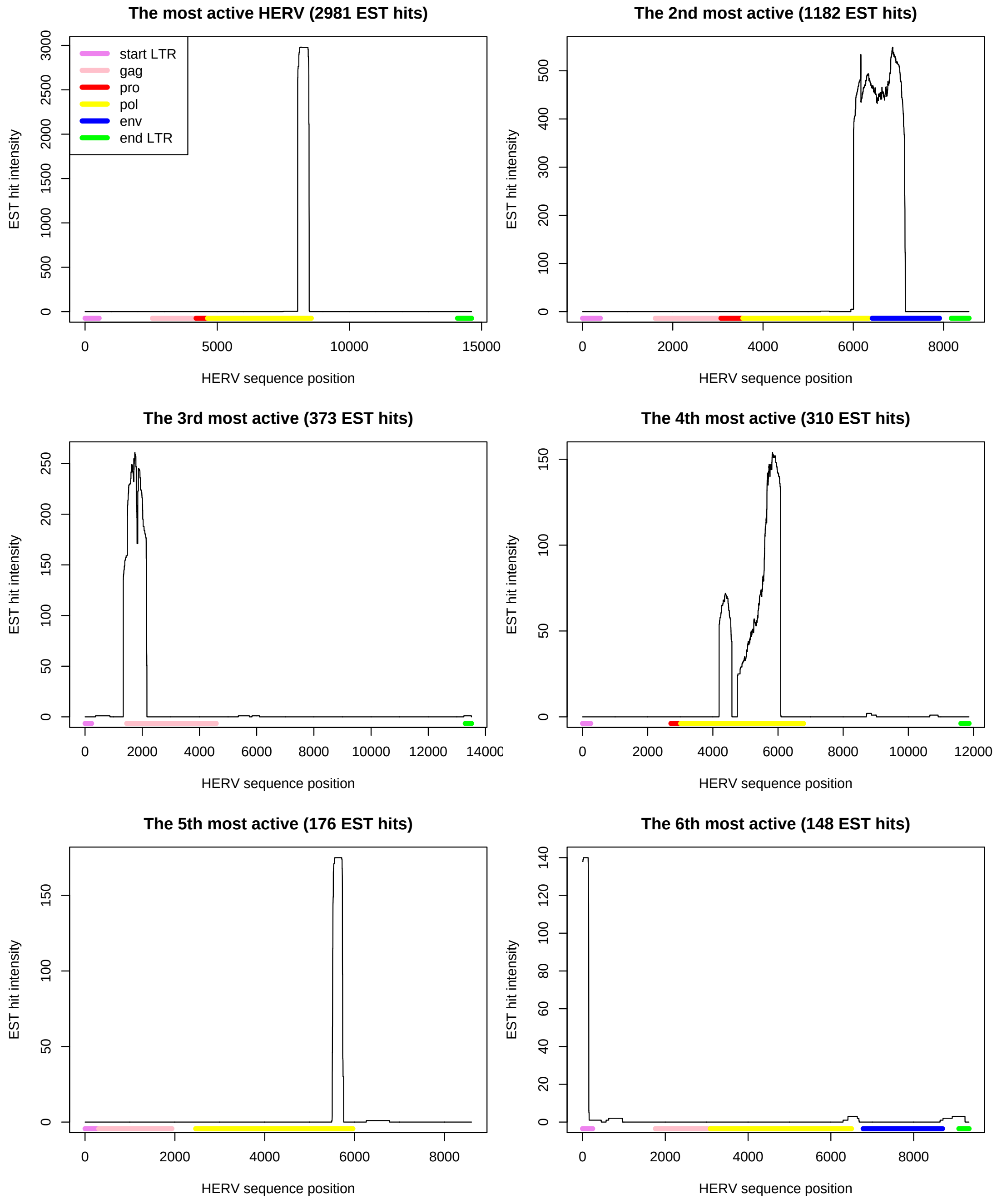

Supplementary Figure 4, continued...

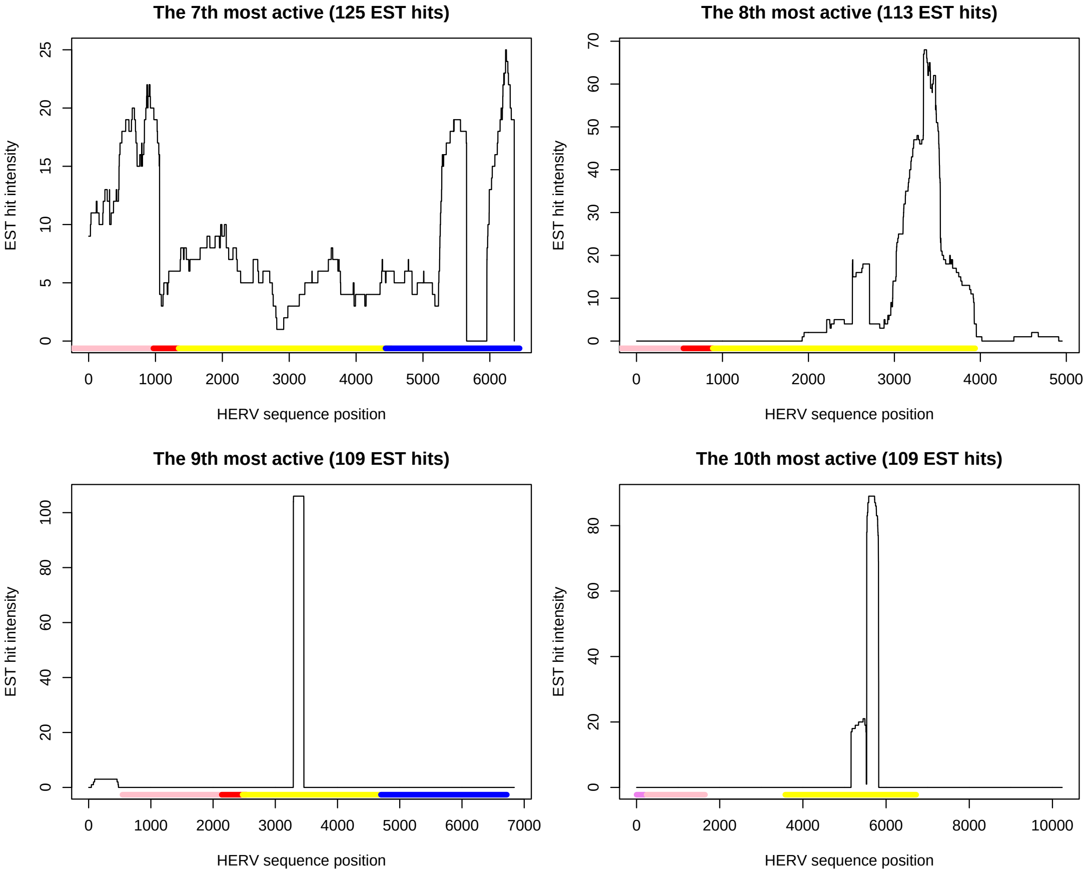

Supplementary Figure 5 - EST hit locations for the syncytin genes

The colored blocks below the curve represent the HERV structure (genes, LTRs) and the curve presents EST hit intensity along the HERV structure. See Table 1 for more information on these HERVs.

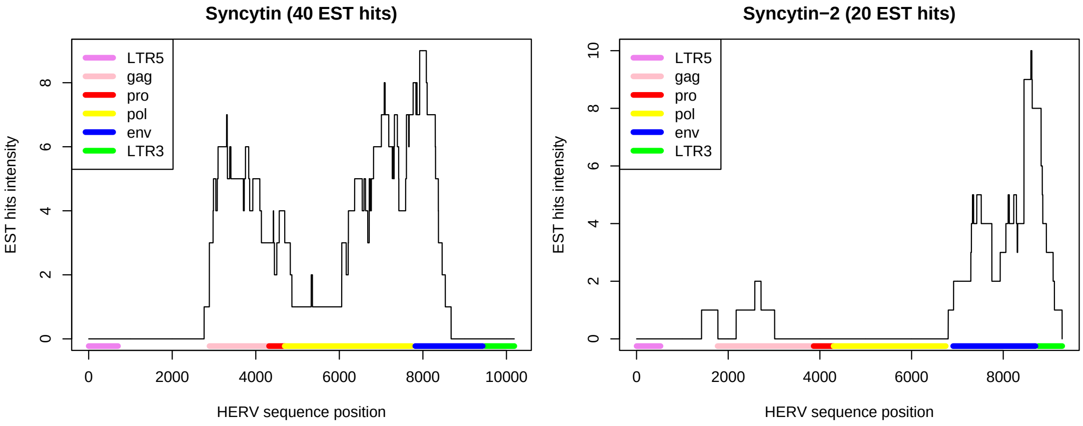

Supplementary Figure 6 - Group activity summary

Left: The overall activity in the group. The group activity is obtained by accumulating the EST counts of its members. *Right:* Box-and-whiskers plot for logarithmic EST counts in the groups. A finer grained figure than Fig. 5, but giving partly similar information. The dashed line is the threshold value for activity. The groups are in size order (See Fig. 5).

HERV-48 family is the most active, but the activity is explained by a single outlier. Also ERV9 has a large portion of its activity explained by one highly active element. In addition to these two, HERV-H, HERV-E, HML-6, HERV-I, HML-2, and HML-3 are the most active groups. All of these have been reported to be relatively active also in earlier studies [3, 4], exceptions are HERV-I and HERV-48 which were not analyzed earlier. In [4] HERV-E was less active than HML2, and in [3] vice versa. Our results support the latter result. We detect also several inactive families, for example HML-5 and HML-7 are inactive. Also the very old family HERV-L is relatively inactive. HML-5 and HERV-L were almost inactive also in [3].

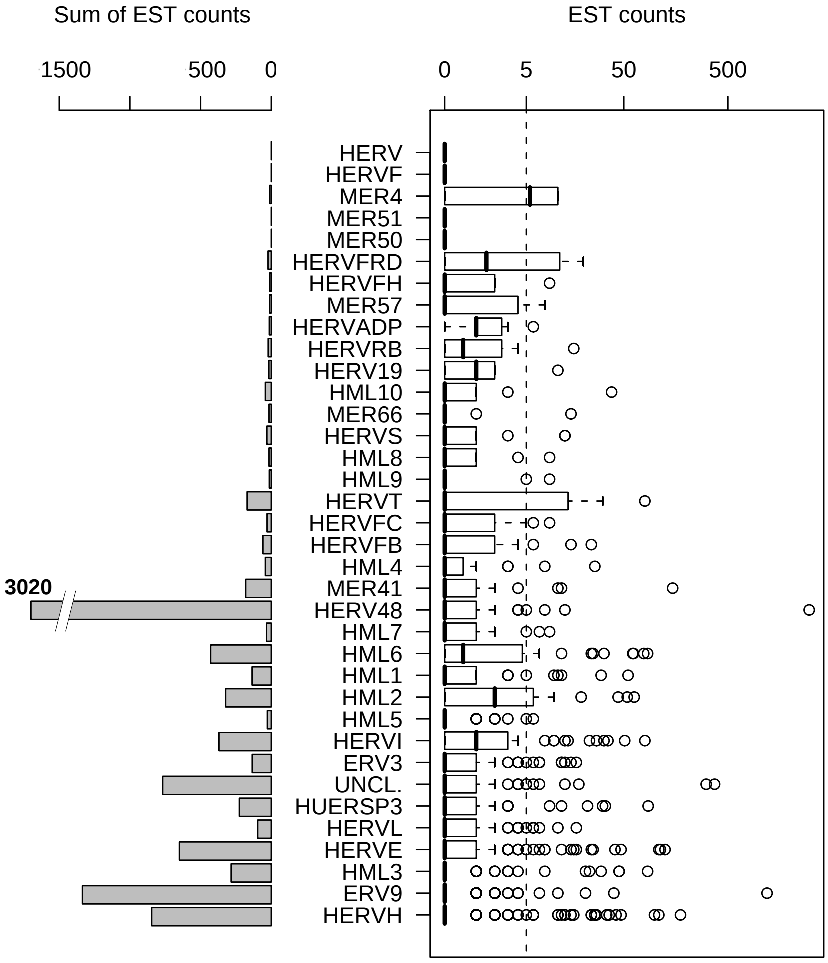

Supplementary Figure 7 - Reliability estimation by resampling

Bootstrap is used to produce a resampling distribution of the activity values of each HERV. The activity learned from complete EST data can then be compared to this resampling distribution. The resampling distribution is used to produce confidence intervals for HERV activities.

Top: The EST sequence data is sampled with replacement to produce a sample data set of the same size as the original EST data set. This is done 10000 times.

Middle: Each sample data set is used to compute the activity values for all HERVs (that are included in that resample). This can be done for both the HMM approach and the BLAST approach, similarly as when learning the activities from the original EST data set. For the HMM-model only the activities are reoptimized; other parameters are kept fixed.

Bottom: example of the resampling distribution for one HERV.

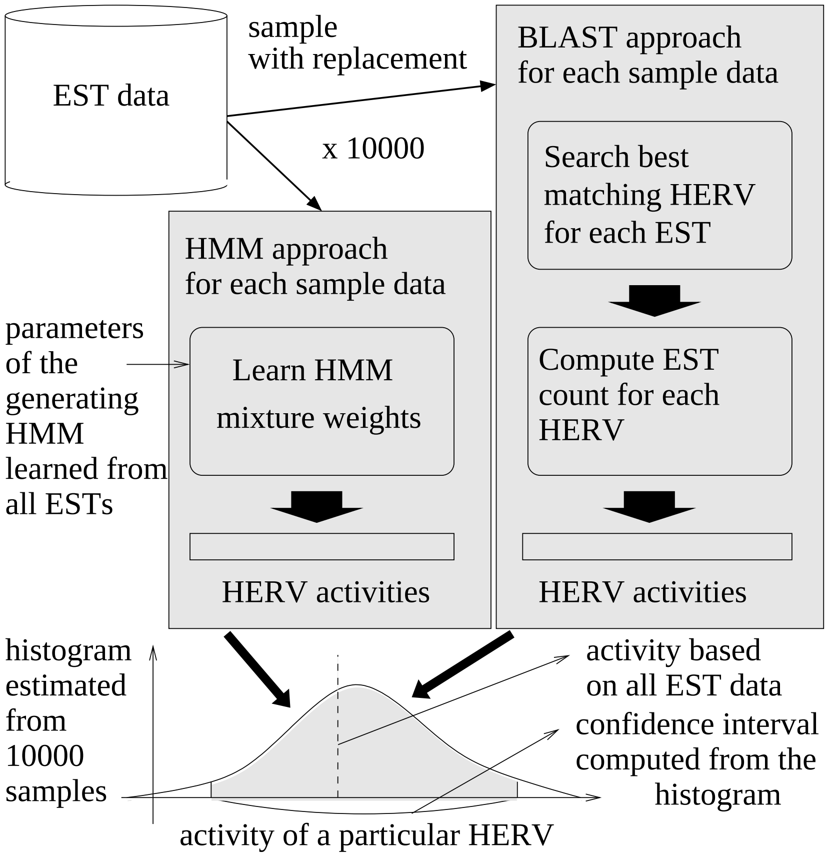

Supplement: Additional File 1 — Supplementary material. contains supplementary details on our methods and supplementary figures 1-7. [file 1471-2105-8-S2-S11-S1.pdf]
